# Anomalies on anterior and posterior complex views in fetuses with partial agenesis of corpus callosum

**DOI:** 10.1002/uog.29292

**Published:** 2025-07-11

**Authors:** F. Viñals, F. Correa, R. García‐Rodríguez, A. Tubau‐Navarra, M. Esparza, L. Diaz

**Affiliations:** ^1^ Ultrasound Department Sanatorio Alemán Clinic Concepción Chile; ^2^ Obstetrics Department, Faculty of Medicine University of Concepción Concepción Chile; ^3^ Fetal and Neonatal Ultrasonography Department Hospital Lusiadas Lisbon Portugal; ^4^ Prenatal Diagnosis Unit, Department of Obstetrics and Gynaecology Complejo Hospitalario Universitario Insular Materno Infantil Las Palmas de Gran Canaria Spain; ^5^ Department of Obstetrics and Gynaecology, Prenatal Diagnosis Unit Hospital Universitario Son Llàtzer Palma de Mallorca Spain

**Keywords:** anterior complex, CC dysgenesis, CNS anomalies, corpus callosum, partial agenesis, posterior complex, ultrasound screening

## Abstract

**Objective:**

The aim of the present study was to describe abnormal anatomical features of the anterior (AC) and posterior (PC) complexes identified during basic ultrasound assessment of the fetal brain in cases diagnosed with partial agenesis of the corpus callosum (pACC).

**Methods:**

This was a retrospective observational study of 22 fetuses diagnosed with pACC between January 2010 and June 2024. The cases were reviewed by fetal neurosonographers who evaluated the morphology of AC and PC structures visualized during basic axial fetal brain screening at 20–33 weeks' gestation in three different referral centers in Chile and Spain. All cases were also assessed independently by another fetal neurosonographer who was blinded to the original reports, using images, videoclips and three‐dimensional volume datasets.

**Results:**

Regarding the structures that comprise the AC, the cavum septi pellucidi had an abnormal appearance in 20/22 cases and the anterior horn of the lateral ventricle had a dysmorphic pattern, characterized by being parallel to the midline, in 11/22 cases. PC abnormalities observed included widening of the interhemispheric fissure posteriorly in 20/22 cases, and absent callosal sulcus with no visualization of the corpus callosum crossing the midline in 19/22 cases. Observation of these PC abnormalities had a very high level of agreement between the original and the independent neurosonographers, ranging between 95% and 100%.

**Conclusion:**

Evaluation of the AC and PC during the basic examination of the fetal brain could significantly improve the prenatal diagnosis of corpus callosum dysgenesis. © 2025 The Author(s). *Ultrasound in Obstetrics & Gynecology* published by John Wiley & Sons Ltd on behalf of International Society of Ultrasound in Obstetrics and Gynecology.

## INTRODUCTION

The corpus callosum (CC) is the largest telencephalic commissure, and owes its formation to a complex and diverse series of processes that determine its four‐segment structure: rostrum, genu, corpus and splenium^1^. The absence of one or more of these segments is called partial agenesis of the corpus callosum (pACC)^2^, ^3^. In contrast to complete agenesis of the CC, in pACC a remnant of the commissural structure is present. However, there is considerable variation in diagnostic criteria when the CC is present but shortened and/or shows alterations in thickness and homogeneity. The diverse nomenclature and our limited understanding of the pathophysiological mechanism based on imaging have prompted the use of the term ‘CC dysgenesis’ to encompass all abnormalities of a visible CC (excluding complete agenesis)^4^, ^5^, ^6^. Moreover, the affected CC segment(s) represents just one feature of an abnormal developmental process, with other cerebral and extracerebral structures also potentially involved, depending on the etiology of the CC anomaly^7^.

Recent data have revealed that when the CC is present but dysgenic in appearance it is not possible to deduce whether there are fibers that fail to cross the midline^8^ or whether the anomaly detected will contribute to predicting normal or abnormal brain function. Therefore, we believe that enhancing the detection rate of CC anomalies would be an important starting point for further understanding.

Visualization of the anterior complex (AC) and the posterior complex (PC) on routine axial sonographic planes has been proposed as a tool for improving the diagnosis of fetal midline anomalies^9^, ^10^. After incorporating visualization of both complexes into our routine ultrasound scans^10^, ^11^, ^12^, we were able to identify some abnormalities in fetuses that were later confirmed to be diagnosed with CC dysgenesis. The aim of this study was to describe the abnormalities visualized in the structures of the AC and PC during basic ultrasound assessment in cases diagnosed with pACC.

## METHODS

### Study population

This was a retrospective multicenter study involving fetuses diagnosed with pACC, evaluated by three fetal neurosonographers (F.V., R.G.‐R., A.T.‐N.) in three referral centers (Sanatorio Alemán Clinic, Concepción, Chile; Complejo Hospitalario Universitario Insular Materno Infantil, Las Palmas de Gran Canaria, Spain; Hospital Universitario Son Llàtzer, Palma de Mallorca, Spain) between January 2010 and June 2024. Inclusion criteria included cases with at least one neurosonographic evaluation via the transabdominal and transvaginal approach, according to the International Society of Ultrasound in Obstetrics and Gynecology (ISUOG) guidelines^13^. All cases were required to have clinical images of the fetal brain in .jpg (still images), .avi (video) and three‐dimensional volume dataset formats, obtained both transabdominally and transvaginally. Genetic (prenatal karyotype analysis, chromosomal microarray analysis and/or exome sequencing) and imaging studies were recorded. Prenatal and neonatal follow‐up (including intrauterine or neonatal death and termination of pregnancy) were reported, and associated anomalies were described. Exclusion criteria included all cases with a diagnosis of complete agenesis of the CC, CC dysraphism^2^, ^3^, ^14^ and CC anomalies associated with interhemispheric cysts and/or severe ventriculomegaly. All cases in which the fetus could not be evaluated transvaginally (for example, non‐cephalic presentation and placenta previa) and did not have complementary fetal magnetic resonance imaging (MRI) were excluded.

**Table 1:** Maternal and fetal characteristics of 22 fetuses diagnosed with partial agenesis of the corpus callosum

Parameter	Value
Maternal age (years)	35.0 (26.7–39.6)
Maternal BMI (kg/m^2^)	26.8 (21.3–33.2)
First fetal NSG at 20 + 0 to 23 + 6 weeks	17 (77.3)
Two or more fetal NSG	12 (54.5)
Fetal and/or neonatal MRI	9 (40.9)
Prenatal karyotype analysis, CMA and/or exome sequencing	17 (77.3)
Intrauterine fetal death*	2 (9.1)
Neonatal death*	1 (4.5)
Termination of pregnancy†	3 (13.6)

Data are given as median (interquartile range) or *n* (%).

* Due to trisomy 18.

† One case each of trisomy 13, Coffin–Siris syndrome and Bohring–Opitz syndrome.

BMI, body mass index; CMA, chromosomal microarray analysis; MRI, magnetic resonance imaging; NSG, neurosonography.

The evaluations were performed at 20–33 weeks' gestation using Voluson E6, E8, E10 or E22 ultrasound systems (GE Healthcare, Zipf, Austria) equipped with transabdominal (RAB 4–8‐MHz or 6D‐MHz) and transvaginal (RIC 5–9‐MHz or 6–12D‐MHz) probes. Clinical and ultrasound data were obtained from the respective databases of each center. 4DView software (version 18Ext.5; GE Healthcare) was used for fetal brain evaluation and measurements.

The fetal brain examination was performed according to the ISUOG guidelines for basic screening examination of the fetal brain as well as those for targeted neurosonography^13^, ^15^. The fetal neurosonographer was asked to review AC and PC structures in their respective cases, based on axial planes defined previously by our group^10^, ^11^, ^12^. To test agreement between the operators, an independent fetal neurosonographer (F.C.), who was blinded to the original reports, also evaluated each case using stored images, videoclips and volume datasets.

Informed consent was obtained from all patients, who gave their permission to collect images and clinical data. The study was approved by the ethics committee of the Faculty of Medicine, University of Concepción, Chile (CEC 21/2022).

### Definitions

**Figure 1:**
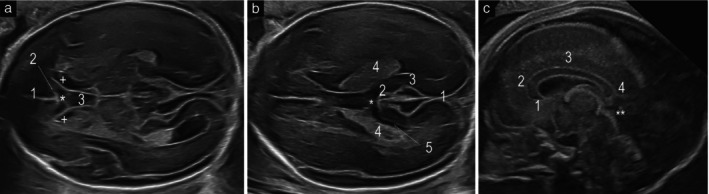
Transabdominal (a,b) and transvaginal (c) ultrasound images in a normal fetus at 22 weeks' gestation. (a) Image at the level of the anterior complex in the axial (transventricular) plane, showing interhemispheric fissure (1), callosal sulcus (2), cavum septum pellucidum (3), genu of the corpus callosum (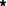) and ‘comma‐shaped’ anterior horns of the lateral ventricles (+). (b) Image at the level of the posterior complex, showing interhemispheric fissure (1), callosal sulcus (2), parieto‐occipital fissure (3), choroid plexus of the lateral ventricle (4) and medial wall of the lateral ventricle (5) in oblique disposition relative to the midline, and corpus callosum (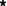). (c) Transvaginal image in midsagittal plane showing all parts of the corpus callosum, including rostrum (1), genu (2), body (3) and splenium (4), with posterior aspect reaching the quadrigeminal cistern (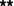).

The appearance of the cavum septum pellucidum (CSP) and its length‐to‐width ratio were evaluated at the level of the transventricular plane, based on definitions reported previously^10^, ^16^. CC segmentation included: the rostrum, which projects posteriorly and inferiorly from the genu; the genu, which forms the anterior part of the CSP; the body, which is the horizontal portion that borders the CSP superiorly; and the splenium, which is the most caudal segment that reaches the quadrigeminal cistern from 20 weeks' gestation (Figure 1). We defined pACC when the CC was present but not fully developed^1^, ^2^, ^3^.

### Statistical analysis

Data are presented as *n* (%) and median (interquartile range), calculated using Excel 2022 (Microsoft Corp., Redmond, WA, USA) and SPSS version 26 (IBM Corp., Armonk, NY, USA). The Shapiro–Wilk test was used to assess normal distribution and the chi‐square test and Fisher's exact test were used to compare categorical variables. Percentage agreement and Cohen's kappa coefficient (κ) were calculated using 2 × 2 contingency tables and were used to assess interobserver agreement between the original neurosonographer and the independent neurosonographer. Cohen's κ and the 95% CI were calculated using the formula 95% CI = κ ± (1.96 × SEκ)^17^, where SEκ is the standard error of κ. The Wilson score interval without continuity correction^18^ was used to calculate 95% CIs for fractions and proportions. *P* ≤ 0.05 was considered statistically significant.

## RESULTS

Of all cases of pACC diagnosed during the study period, 22 fulfilled all the inclusion criteria, of which 14 were from the referral center in Concepción, Chile, five from the center in Las Palmas de Gran Canaria, Spain, and three from the center in Palma de Mallorca, Spain. Maternal and fetal characteristics are summarized in Table 1. Most of the cases (16/22) were evaluated from 2020 onwards, with only six cases evaluated in previous years, dating back to 2010.

**Figure 2:**
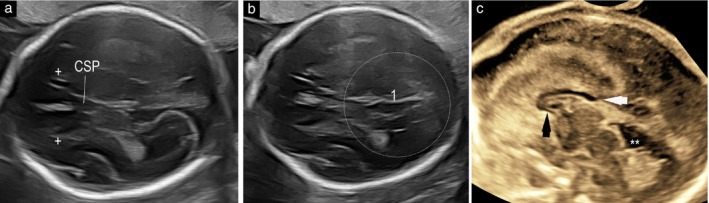
Transabdominal (a,b) and transvaginal (c) ultrasound images in a fetus with partial agenesis of the corpus callosum at 23 weeks' gestation. (a) Image at the level of the anterior complex demonstrating normal triangular appearance of cavum septum pellucidum (CSP) with length‐to‐width ratio of ≥ 1.5. (b) Image at the level of the posterior complex in axial plane demonstrating that the callosal sulcus is absent, parieto‐occipital fissure lacks definition (1) and corpus callosum does not cross the midline. (c) In the midsagittal plane, both the rostrum (black arrowhead) and part of the splenium (white arrowhead) were missing on direct three‐dimensional transvaginal visualization. Arrows indicate where these structures should be observed. 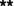, quadrigeminal cistern; +, anterior horn of the lateral ventricle.

Ultrasound findings at the level of the AC are provided in Table 2. In 2/22 cases, the CSP had a normal triangular or quadrangular aspect that was longer in the anteroposterior direction, at the level of the axial transventricular plane. In the other 20 cases, the CSP had a length‐to‐width ratio of < 1.5 (Figure S1). In both cases with a normal CSP appearance, the CC rostrum and part of the splenium were found to be missing in the midsagittal view; one of these cases is depicted in Figure 2. There was 100% agreement between the original neurosonographer and the independent neurosonographer concerning the normal appearance of the CSP. In one case (1/22 (4.5%)), there was no concordance in the definition of the appearance of the anterior horns of the lateral ventricles (AH) between the original neurosonographer and the independent neurosonographer.

**Table 2:** Ultrasound findings at the level of the anterior complex in 22 fetuses diagnosed with partial agenesis of the corpus callosum

Parameter	Value
Segment of corpus callosum absent	
Rostrum + genu + part of splenium	10 (45.5)
Rostrum + part of splenium	9 (40.9)
Part of splenium	3 (13.6)
Normal appearance of CSP	2 (9.1)
Abnormal appearance of CSP	20 (90.9)
Length‐to‐width ratio of CSP	
≥ 1.5	2 (9.1)
< 1.5	20 (90.9)
AH appearance	
Comma‐shaped (normal)	11 (50.0)
Parallel to midline	11 (50.0)
GA at imaging in cases of parallel AH (weeks)	22.7 (21.3–26.1)
< 24 weeks	7/11 (63.6)

Data are given as *n* (%), median (interquartile range) or *n*/*N* (%).

AH, anterior horn of the lateral ventricle; CSP, cavum septum pellucidum; GA, gestational age.

**Figure 3:**
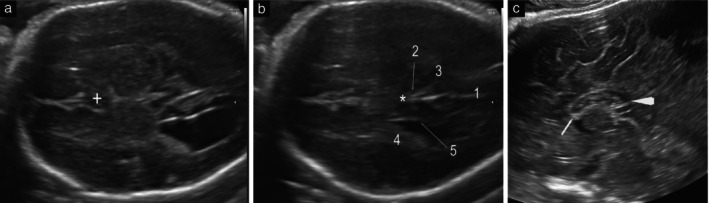
Transabdominal (a,b) and transvaginal (c) ultrasound images in a fetus with partial agenesis of the corpus callosum at 27 weeks' gestation. (a) Image at the level of the anterior complex in axial plane demonstrating abnormal appearance of cavum septum pellucidum (+) with length‐to‐width ratio of < 1.5. (b) Image at the level of the posterior complex in axial plane demonstrating normal appearance of interhemispheric fissure (1), callosal sulcus (2), parieto‐occipital fissure (3) and the corpus callosum crossing the midline (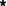); choroid plexus of the lateral ventricle (4) is present and medial wall of lateral ventricle is parallel rather than oblique to the midline (5). (c) In the midsagittal plane, both the rostrum and part of the genu (arrow) and the splenium (arrowhead) were missing. Arrows indicate where these structures should be observed.

Ultrasound findings at the level of the PC and the agreement between neurosonographers are shown in Table 3. PC abnormalities observed included widening of the interhemispheric fissure posteriorly in 20/22 cases, and absent callosal sulcus with no visualization of the corpus callosum crossing the midline in 19/22 cases. In 3/22 cases, both the presence of the callosal sulcus and the CC crossing the midline were visible at the level of the PC, and in two of these cases the interhemispheric space was considered normal. However, in all three of these cases, the CSP appearance and length‐to‐width ratio were abnormal during evaluation of the AC (Figure 3). Observation of these PC abnormalities had a very high level of agreement between the original and the independent neurosonographers, with an overall agreement of 98% (Table 3).

**Table 3:** Ultrasound findings at the level of the posterior complex in 22 fetuses diagnosed with partial agenesis of the corpus callosum, and agreement between original and independent fetal neurosonographers

Parameter	*n*/*N* (%)	Agreement betweenobservers (% (95% CI))	Kappa (95% CI)	*P*
Callosal sulcus and corpus callosum not seen	19/22 (86.4)	100 (91.9–100)	1.0 (1.0–1.0)	0.001
Increase in interhemispheric space		95.4 (78.2–99.2)	0.77 (0.35–1.00)	0.013
Original neurosonographer	20/22 (90.9)			
Independent neurosonographer	19/22 (86.4)			
Overall agreement	—	98.4 (91.9–99.7)	0.93 (0.8–1.0)	0.004

## DISCUSSION

There are two major challenges in the prenatal diagnosis of pACC from a diagnostic imaging perspective. The first challenge relates to screening. It is well known that basic sonographic axial screening of the fetal brain limits the diagnosis of CC malformations, as the midsagittal view of the CC is the gold standard for diagnosing CC anomalies^2^, ^7^, ^14^. In pACC, since the CC is present but abnormal in appearance, a direct view of this structure naturally provides better diagnostic sensitivity and specificity. However, this approach is contrary to the principles governing our current screening procedures, which recommend maintaining a transabdominal ultrasound examination that is applicable universally to the entire low‐risk pregnant population^15^, ^19^.

The second challenge arises at the diagnostic stage of the screening pathway^19^, when advanced imaging modalities such as multiplanar fetal neurosonography and fetal MRI are used. These modalities face additional difficulties, including the profound heterogeneity in the nomenclature and definitions of pACC. For example, a recent systematic review found nine different definitions for prenatal pACC, including any part of the CC missing, a specific part of the CC missing and even a ‘short‐for‐gestational‐age but complete CC’ definition, as well as overlap in definitions, with the same pathology given varying names in different studies^6^. There is also substantial heterogeneity in the methods and biometric values used to define normality based on quantifying the size of the CC, despite our limited understanding of how normal CC size can vary among individuals^4^, ^5^, ^20^, ^21^. In our study, a fully developed CC was defined as having four segments and extending caudally to reach the level of the quadrigeminal cistern from 20 weeks onwards. We defined pACC as when the CC was present but not fully developed^1^, ^2^, ^3^. This definition implies optimal diagnostic resolution, obtained by transvaginal multiplanar fetal neurosonography and fetal MRI^13^.

pACC is a heterogenous condition that can be caused by developmental arrest of any portion of the CC or by a disruptive event (infection, vascular insult, toxic agent). It can be isolated or part of a polymalformative or genetic disease.

As reported in previous studies, the axial plane is insufficient to screen for CC dysgenesis^2^, ^7^. Previously, the suspicion for CC anomalies (especially complete absence of the CC) has been based primarily on the absence of the CSP and on the presence of colpocephaly in the axial view. Over the last 10 years, our group has used and reported on a series of precise landmarks combined with imaging in different axial planes to improve the screening for supratentorial anomalies in the low‐risk population. The landmarks are grouped into those of the AC and those of the PC. We defined the normal appearance of the CSP to be quadrangular or triangular with an anterior base, and longer in its length than in its width, at the level of the transventricular plane^9^, ^10^, ^11^. This subjective observation is made on the same basis as the objective evaluation proposed by Karl *et al*.^16^ on the same axial (transventricular) plane. For the cases included in the present study, at the level of the AC there was 100% agreement between the original neurosonographer and the independent neurosonographer for the defined appearance and length‐to‐width ratio of the CSP, which were abnormal in 20/22 (90.9%) cases, thus proving to be a valuable indicator for the prenatal suspicion of pACC^15^ (Figure S2). This discrepancy with previous reports is possibly due to a lack of a precise definition of the normal appearance of the CSP and because it was evaluated in a different plane to the transventricular plane^7^, ^22^. Additionally, while evaluating the AC, a dysmorphic pattern of the AH was observed in 11/22 (50%) cases; this was characterized by absence of the normal comma shape and by one AH described to be parallel to the other AH and to the midline. The lateral ventricles are commonly found to be parallel to each other and to the midline in complete ACC, and this feature is also reported to be present to variable degrees in pACC^13^.

The CC develops first in the region of the anterior body and grows bidirectionally to form the genu and the rostrum at the anterior portion, as well as the posterior part of the body and the splenium^23^. In our cases and in the literature^7^, the posterior segments of the CC are usually missing in pACC. Therefore, efforts must be made to improve visualization of the posterior portion, which is partially identifiable in the axial plane of the PC.

To visualize the PC, the sonographic plane can be achieved by slicing cranially from the transventricular plane until the interhemispheric fissure is interrupted by the crossing of the CC in front of the parieto‐occipital fissure^9^. In previous studies, we found at least a 95% visualization rate for the structures that comprise the PC, with significant agreement rates between expert and non‐expert examiners in neurosonography, for the subgroup evaluated at 20 + 0 to 23 + 6 weeks' gestation^10^. Specifically, visualization of the CC crossing the midline at the level of the PC was achieved in 97% of cases, with a high level of agreement between the expert and non‐expert examiners in second‐trimester scans^10^. In the present study, for 19/22 (86.4%) cases of pACC, neither the callosal sulcus nor the CC was seen at the level of the PC, with 100% agreement between neurosonographers. In the remaining three cases, two had an interhemispheric space and a parieto‐occipital fissure of normal appearance at the level of the PC. However, in all three of these cases, the appearance and the length‐to‐width ratio of the CSP were abnormal at the level of the AC. Upon analyzing the morphology of the CC in the midsagittal plane, the rostrum and part of the splenium were absent in two of these three cases.

The PC has proved to be a valuable tool in the assessment of the posterior midline during basic fetal brain screening. In fact, in this study, when the anatomical abnormalities seen in both complexes were evaluated, only one case of CC anomaly had a normal AC but abnormal PC, which was a case of pACC with a missing splenium. Visualization of the PC also enables evaluation of the position of the choroid plexuses and lateral ventricles; in cases of complete ACC, these structures do not present their normal oblique position and instead become parallel. In cases of pACC, these structures assume a variable position between oblique and parallel^10^, ^14^.

### Limitations

We acknowledge several limitations of our study. Firstly, it was retrospective in nature with a small sample size, possibly owing to the rarity of pACC and the strict case‐selection criteria. Since pACC is an uncommon anomaly, multicenter studies are needed to ensure inclusion of a larger number of patients. Secondly, the CC in some of the included cases had, in addition to partially absent segments, a somewhat heterogeneous appearance and/or suspected abnormal thickness. This demonstrates the limitations of an imaging‐based diagnosis and the potential convenience of using the term CC dysgenesis to describe these cases, rather than defining them as pACC. Thirdly, it was not possible to determine whether the expert findings detected in this study would be identified during non‐expert screening. Although previous studies have demonstrated that visualization of both complexes can feasibly be included in screening^10^, ^11^, ^12^, with a high level of agreement between expert and non‐expert operators, further prospective studies are needed to confirm the usefulness of incorporating AC and PC evaluation for improving the detection of CC dysgenesis.

### Conclusion

This study shows that evaluation of the AC and PC may improve the performance of fetal brain ultrasound screening, enhance detection of potential CC dysgenesis and facilitate subsequent referral to tertiary care, and thus ultimately result in better management of prenatal cerebral anomalies. Further prospective studies are needed to confirm our findings.

## Supporting information


**Figure S1** Individual measurements of length‐to‐width ratio of the cavum septi pellucidi (CSP ratio) in 22 fetuses with partial agenesis of the corpus callosum. Two cases had normal CSP ratio (circle) and 20 were below the reference cut‐off of 1.5 (triangle), in relation to biparietal diameter (BPD) in mm.

**Figure S2** Transabdominal ultrasound images in axial transventricular plane at the level of the anterior complex (arrow) in fetuses with agenesis of one or more segments of the corpus callosum at 21 (a), 22 (b,c), 23 (d–f), 26 (g), 27 (h), 28 (i) and 33 (j) weeks' gestation.

## Data Availability

The data that support the findings of this study are available from the corresponding author upon reasonable request.
