# Supplementary figures and images for: Anomalies on anterior and posterior complex views in fetuses with partial agenesis of corpus callosum
Source: Ultrasound Obstet Gynecol. 2025 Jul 11;66(3):347–52. doi: 10.1002/uog.29292 (PMC12401505; doi:10.1002/uog.29292)

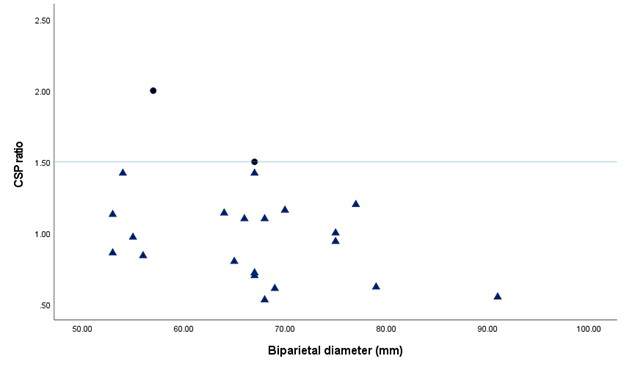

Supplement: Supplementary file 1 — Figure S1 Individual measurements of length‐to‐width ratio of the cavum septi pellucidi (CSP ratio) in 22 fetuses with partial agenesis of the corpus callosum. Two cases had normal CSP ratio (circle) and 20 were below the reference cut‐off of 1.5 (triangle), in relation to biparietal diameter (BPD) in mm. [file UOG-66-347-s001.tif]

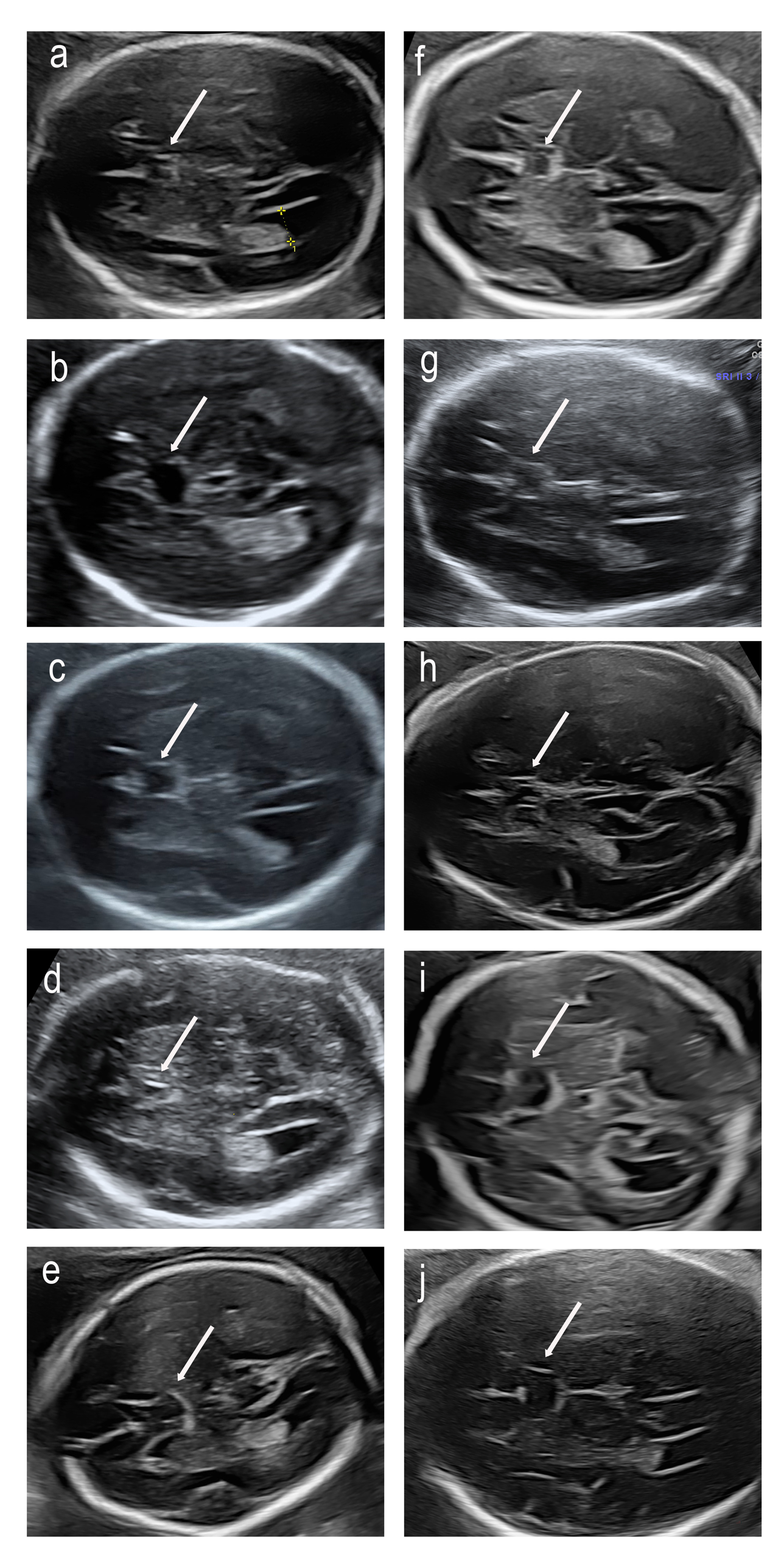

Supplement: Supplementary file 2 — Figure S2 Transabdominal ultrasound images in axial transventricular plane at the level of the anterior complex (arrow) in fetuses with agenesis of one or more segments of the corpus callosum at 21 (a), 22 (b,c), 23 (d–f), 26 (g), 27 (h), 28 (i) and 33 (j) weeks' gestation. [file UOG-66-347-s002.jpg]
